# Understanding tissue-resident macrophages unlocks the potential for novel combinatorial strategies in breast cancer

**DOI:** 10.3389/fimmu.2024.1375528

**Published:** 2024-07-22

**Authors:** Manjusha Biswas

**Affiliations:** ^1^ Department of Molecular Biomedicine, Developmental Biology of the Immune System, Life & Medical Sciences (LIMES) Institute, University of Bonn, Bonn, Germany; ^2^ Institute of Pharmacology and Toxicology, University Hospital, University of Bonn, Bonn, Germany

**Keywords:** breast cancer, tissue resident macrophage, macrophage ontogeny, tumor microenvironment, immunotherapy

## Abstract

Tissue-resident macrophages (TRMs) are an integral part of the innate immune system, but their biology is not well understood in the context of cancer. Distinctive resident macrophage populations are identified in different organs in mice using fate mapping studies. They develop from the yolk sac and self-maintain themselves lifelong in specific tissular niches. Similarly, breast-resident macrophages are part of the mammary gland microenvironment. They reside in the breast adipose tissue stroma and close to the ductal epithelium and help in morphogenesis. In breast cancer, TRMs may promote disease progression and metastasis; however, precise mechanisms have not been elucidated. TRMs interact intimately with recruited macrophages, cytotoxic T cells, and other immune cells along with cancer cells, deciding further immunosuppressive or cytotoxic pathways. Moreover, triple-negative breast cancer (TNBC), which is generally associated with poor outcomes, can harbor specific TRM phenotypes. The influence of TRMs on adipose tissue stroma of the mammary gland also contributes to tumor progression. The complex crosstalk between TRMs with T cells, stroma, and breast cancer cells can establish a cascade of downstream events, understanding which can offer new insight for drug discovery and upcoming treatment choices. This review aims to acknowledge the previous research done in this regard while exploring existing research gaps and the future therapeutic potential of TRMs as a combination or single agent in breast cancer.

## 1 Introduction

Breast cancer is currently the most commonly diagnosed cancer worldwide, surpassing lung cancer in 2020. The International Agency for Research on Cancer (IARC) and partner institutions predict breast cancer cases will rise to 40% by 2040 with an increase of 50% mortality rate worldwide, leading to more than one million deaths per year (1). Various chemotherapy regimens and targeted therapy options are available to treat breast cancer, but immunotherapy is still evasive (2). However, the presence of tumor-infiltrating lymphocytes, identification of different innate immune subsets, and transcriptomics analysis identifying immune gene signatures suggest the potential of combining immunotherapy with standard of care (SOC) (2–4). While the role of immune checkpoint inhibitors, bispecific antibodies, CAR-T cell therapy, etc. have been recognized across various solid tumors including breast cancer (5–7), the innate immune cells, particularly macrophages, are emerging as novel candidates for combination therapy. To exploit macrophage’s potential as a therapeutic target in breast cancer, understanding their ontogeny is crucial as developmental origins dictate their functional commitment in steady state and disease. Single-cell RNA sequencing (scRNA-seq) data indicating existence of macrophage phenotypes beyond the conventional M1 and M2 spectrum (8) inspires a deeper dive into the subject. Ontogenically, macrophages can be either resident or recruited. Tissue-resident macrophages (TRMs) being the guardians of homeostasis garner more attention and also focus of this review. As startling new data have appeared on TRM diversity, their potential mechanistic role in breast cancer, and their influence on varied cellular or stromal partners in breast tumor microenvironment (TME), the complex crosstalk is reviewed and the rising therapeutic scope of resident macrophages in breast cancer as single mechanistic target or a potential candidate for combination therapy is assessed.

## 2 Tumor immune microenvironment in breast cancer

**Figure 1:**
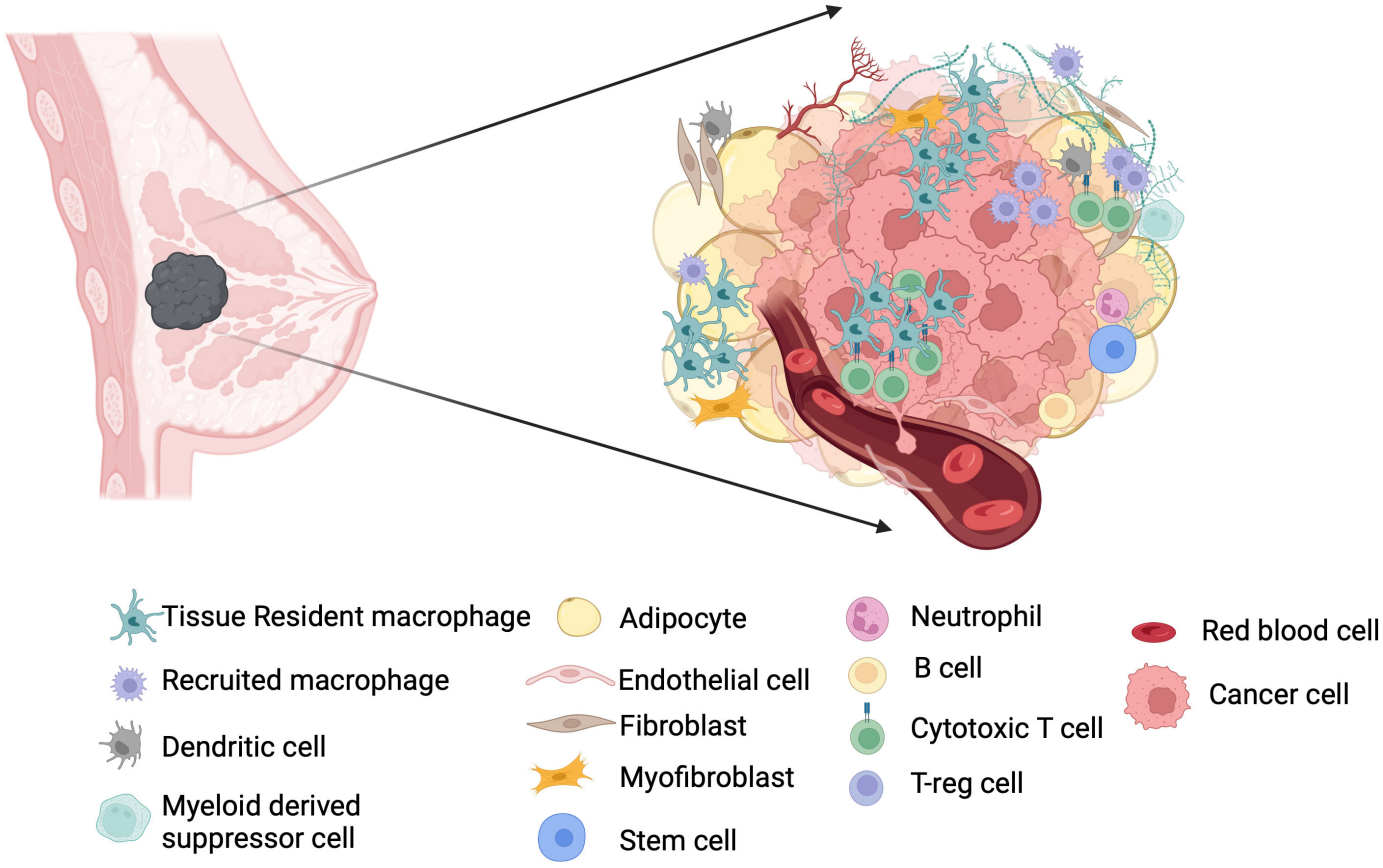
TME in breast cancer. Microenvironment in breast cancer is formed by multiple cell types, and their crosstalk is implicated in the pathogenesis of breast cancer. Resident macrophages predominate the breast TME along with T cells and help in disease progression or attenuation depending on their heterogeneity not only by interacting tumor cells and T cells but also with other stakeholder cells such as fibroblasts and adipocytes. Tumor associated macrophages contain both resident and recruited macrophages.

The breast cancer microenvironment is intricate (**Figure 1**). Apart from tumor cells, it has different stromal cells and extracellular matrices (ECMs) (9, 10). Moreover, genome-wide profiling identifies multiple phenotypes within the major cell types of breast TME (10–12). The myeloid population of the breast TME consists of monocytes, dendritic cells, and macrophages, spceially tumor associated macrophages (TAM) (12). The lymphoid components predominantly include T cells, with several internal phenotypes and B cells with predominance of memory B cells (10, 12, 13). The most common mesenchymal cells in breast TME are fibroblasts, followed by other minor groups like pericytes, endothelial cells, adipose-derived stromal cells, and mesenchymal stem cells (14). Myeloid-derived suppressor cells (MDSCs), a distinct state of differentiation within neutrophil and monocyte lineage, also exist in breast cancer patients (15). At the molecular level, the factors involved in pyroptosis pathways, ferroptosis-related genes, and hypoxia-related genes (HRGs), and cuproptosis-related genes (CRGs) have been recently explored to identify overall survival, immunogenicity, and immune checkpoints in breast cancer microenvironment (16–18). Breast microbiota represents another intriguing new member of the TME (19), but the crosstalk of breast microbiome with cellular and stromal partners in breast cancer remains to be elucidated.

TIMER2.0 database can be used to assess the contribution of immune cell infiltration in tumor progression and regression using multiple computational algorithms modules (20). Large-scale cytometry profiling pinpoints that T cells and macrophages are the most abundant immune cells in the breast TME (10). Single-cell RNA sequencing confirms this fact and also shows that compared to normal breast tissue, TME can have more cytotoxic T cells and activated macrophages (11).

Within the macrophage population, two dominant phenotypes M1 and M2 were described earlier by the macrophage polarization model. However sc-RNAseq data hint toward a wider phenotypic diversity (11). Indeed, CITE-Seq highlighted novel PD-L1/PD-L2^+^ macrophage populations associated with specific disease outcomes (11). Interestingly, Rac/Cdc42i- inhibited macrophages were found to induce an antitumor TME by affecting IL6 secretion and by inhibition of metastatic cancer cells (21). Understandably, macrophage phenotyping in breast TME is not well defined and their mechanism of action in promoting or attenuating the disease is not fully elucidated. It is rather shadowed under the term “tumor associated macrophage” or “TAM”. This allows researchers to explore breast macrophages in light of developmental heterogeneity and functional commitment. Once the full spectrum of macrophage phenotypes is revealed, their interaction with other key components of breast TME will pave the way for new combinatorial approaches.

## 3 Developmental heterogeneity of macrophage in breast

Macrophage heterogeneity is not yet fully understood (22–24), but their developmental origin certainly plays a role. Tissue-resident macrophages (TRMs or Mϕ) originate from the yolk sac and self-maintain in the peripheral tissue niche. Fate mapping studies show that macrophages develope asynchronously via multiple waves and at different anatomical locations (25, 26). While the first wave can generate primitive microglia, the majority of the tissue-resident macrophages originates in the second wave from erythroid-myeloid progenitor (EMP) through a core macrophage transcriptional program (25, 27). It is important to understand that pMac- and EMP-derived monocytes are two independent progenitors in the developmental trajectory from EMP to long-lived TRMs (25). Hematopoietic stem cells (HSCs) develop as the third wave and migrate via the fetal liver to the bone marrow, where they persist and generate monocytes. These monocytes are constantly recruited in the tissues giving rise to the recruited macrophages (25). The challenge remains due to our limited knowledge about sub-phenotypes of TRM, making their separate anatomical niche within a particular organ, including the breast.

Normal mammary gland (MG) derives from the ectoderm in E10.5, and the F4/80^hi^ and F4/80^int^ macrophage phenotypes are identified in E16.5 (28). Cell fate mapping and antibody depletion studies proved that YS-derived macrophages are F4/80^hi^ in MG, persist lifelong, and express canonical macrophage markers (CD64, MerTK, CD206, C1qa, CSfr1, and Spi) but lack dendritic cell markers (CD11c, Zbtb46, and Itgax) (28, 29). The second population of F4/80^int^ macrophages is fetal liver derived, which contributes to the Mϕ pool postnatally. Mass cytometry data on mammary glands from 3-month-old mice show predominantly F4/80^hi^ CD64^hi^ Siglec-1^hi^ CD206^hi^ TRMs, while CD206^lo^ macrophages were deemed BM derived (28). Another study by Dawson et al., using flow cytometry, showed three Mϕ (CD64^+^ F4/80^+^MerTk^+^) populations, such as CD11c^lo^ CD11b^+^ MHCII^hi^ (Mϕ1), CD11c^lo^ CD11b^+^ MHCII^lo^ (Mϕ2), and CD11c^+^ CD11b^lo^ Ly6C^−^ (Mϕ3). These Mϕs express Lyve1 and CD206 to various extents and prefer either nerve or vessel-associated niches (24). These findings support that heterogeneous TRM populations exist within the breast. When compared within the whole breast, Mϕ3 was enriched in the ducts fourfold and was absent in the fat pads cleared of epithelium. Therefore, Mϕ3 represents a unique entity called ductal macrophage (DM) close to the ductal epithelium, enriched for lysosomal genes, matrix metalloproteinase genes, and notch signaling. Their unique expression of Cx3cr1 confirmed their residential nature (24, 27).

The myeloid compartment of breast tumor profiled in transgenic mouse models showed an increase in Mϕ3 (DM) expanding throughout the tumor and a decrease in the adipose-rich stromal TRM (Mϕ1 & Mϕ2). DM-like TAMs suppress cytotoxic T-cell activity and tumor progression (24). Lavrion et al. also demonstrated stromal and ductal TAMs by scRNA-seq and imaging (30). Stromal I Mϕs were (CD11b, CD206, MHC II, and CCR2 positive) located in adipose tissue stroma or adipose islets, and stromal II Mϕs were located in the connective tissue. Ductal Mϕs were elongated, intraepithelial, and parallel to the basement membrane in steady state and surrounded the TME in breast cancer. They showed significant heterogeneity in CD11b and MHCII expression (30). Interestingly, further scRNA-seq from the sorted myeloid components of the tumor identified expression of Trem2, Cadm1, Folr2, and Mrc1, supporting that both TRM and recruited macrophage build the TAM pool (30). These findings were recapitulated in human breast tumors upon analyzing a published sc-RNAseq database (30, 31). TRMs have been associated with BRCA1-associated human breast cancer tissues; however, their significance is not explicated (31). In addition, ER^+^ cancers are associated with infiltration of TRMs, while HER2^+^ and triple negative cancers are TRM poor (31), which speculates a possible combination of TRM suppression with hormonal therapy. It is conceivable that in the breast TME, various TRM phenotypes exhibit pro- and anti-tumorigenic activity (32, 33).

## 4 Resident macrophage reprogramming in cancer progression

In a healthy breast, TRMs play crucial mechanistic roles. They facilitate phagocytosis of apoptotic epithelial cells during puberty and alveolar cells during involution, while also organizing the structure of terminal end bud and ECM (24, 34–36). Locally active TRMs are the major regulator of branching morphogenesis during breast development (24, 37, 38). Conventionally, macrophages in cancer are termed “tumor-associated macrophages” or TAMs, originating from both resident and recruited pools (30, 39–42). It is suggested that tumors reprogram normal epithelium to produce DM-like TAMs (24, 27). Epigenetic reprogramming of TAMs arising from TRMs by DNA methylation results from tumor-directed perturbation, leading to modulation of several ligands and transcription factors, and this is distinct from monocyte-derived macrophage modulation in the TME (43). In a steady state, TRMs maintain breast tissue homeostasis and anti-tumor immunity in a CSF-1-dependent manner. They form a part of the stem cell niche as supported by studies in Csf1^op/op^ mice (34, 44, 45). Indeed, CSF-1 response signatures are found in 25% of breast cancers, which marks the activation of reprogrammed TRMs and is associated with high tumor proliferation and higher grades (45, 46). Furthermore, altered HIF1a signaling can be another reprogramming mechanism, as hypoxia and anaerobic glycolysis induce TRMs to release growth factors and inflammatory cytokines like TGFß, IL-10, TNF-α, and CCL-8 to promote tumor growth and plasticity, tumor cell adherence, angiogenesis, and metastasis (9, 47–51). The tumor-derived exosomes can reprogram TRMs through TLR-2 and activate MYD88 and NF-kB signaling, inducing increased glycolysis and lactic acidosis, which leads to increased PD-L1 expression and immunosuppression (48, 52). This is also supported by HIF1a-mediated lactate-induced arginase expression in macrophages, leading to tumor progression by cell proliferation (50). Resident macrophages promote disease progression by ECM remodeling (41, 42).Tumor nest macrophages are correlated with microvascular density (53), suggesting their role in neo-angiogenesis. TRMs can also increase hormone resistance by activating the PI3K/Akt/mTOR signaling pathway (14, 54). Macrophages can upregulate PD-L1 expression in multiple solid cancers including breast cancer to modulate cytotoxic T-cell activity (55–57). DM-TAMs showed STAT3 expression associated with immunosuppression (30). MϕTAMs are susceptible to chronic inflammation in obesity and upregulate aromatase expression in obese patients in an IL-6-dependent manner, facilitating the development of ER^+^ breast (58, 59). Interestingly, FOLR2^+^-resident macrophages locally cohabit with CD8^+^ T cells and tertiary lymphoid structures and activate T-cell-mediated cytotoxicity instead of immunosuppression (60). Resident macrophages may initiate the recruitment of HSC-derived macrophages for tumor progression by presenting antigens (24, 61). Zeng et al. showed that TAM-secreted CCL18 reprograms breast-resident fibroblast to a CD10^+^GPR77^+^cancer-associated fibroblast (CAF), which induces chemoresistance through activated NF-kB signaling (62). However, they did not specify the TAM’s developmental nature.

## 5 Resident macrophages in cancer metastasis

Breast cancer can disseminate even when the tumor is regarded *in situ* by light microscopy (63, 64). Metastatic breast cancer is a fairly incurable disease with 5 and 10 years survival rates of approximately 27% and 13%, respectively (65, 66). TRMs play an important role in conditioning premetastatic niche to promote breast cancer metastasis and colonization. Wnt/β-catenin signaling pathway is one of the transcriptional regulators of TRMs. Macrophage-derived WNT-7b ligand is implicated for lung metastasis and TNFa-mediated pro-metastatic environment in breast cancer (26, 67). The perivascular macrophages help tumor cell intravasation. Studies on Csf1^op/op/^PyMT mice show a reduction in circulating tumor cells upon reduction in perivascular macrophage density (68). It is suggested that CD206^hi^ intraepithelial Mϕs (24, 63) produce Wnt-1 causing E-cadherin junction disruption in a CCL2-dependent manner. Subsequently, stromal Mϕs infiltrate into the epithelium leading to early dissemination and subsequent metastasis, especially in HER2^+^ cancer (63). A study in B6 green fluorescent protein (GFP)-transgenic mice with TNBC shows that FOLR2^+^ tissue-resident macrophages dominate the TME (42%–49%), and treatment with clodronate liposomes (which induces apoptosis in macrophages) in a local recurrence model prevented lung and liver metastasis in TNBC (69). Breast cancer nodal metastases are associated with the TIE2^+^ CD31^+^ breast macrophage subset (70), suggesting their residential origin (71). On the contrary, breast cancer nodal metastasis can also be associated with nodal CD169^+^-resident macrophages, which often show adjacent PD-L1 expression and better prognosis (72). Intriguingly, metastatic TME of breast cancer is influenced by resident macrophages of the metastatic organ. A study using humanized and genetic mouse models showed that microglia in the brain orchestrate proinflammatory and tumor-suppressive roles in breast cancer brain metastasis. Animals without microglia were susceptible to increased metastasis, poorer survival, and hampered natural killer and T-cell responses (73). On the contrary, osteoclasts confer resistance to breast cancer cells to DNA damage therapy by enhanced glutamine production in bone metastasis (74). ß-Catenin activation in alveolar macrophages leads to a transcriptional programming enriched for inflammatory, vascular development, cytokine, and chemotactic pathways facilitating lung metastasis (67).

## 6 TNBC and resident macrophages

TNBC is defined as the absence of ER, PR, and HER2 expression and is associated with a high recurrence rate and poor overall survival. High dimensional single-cell profiling of human BRCA-1-associated TNBC shows that macrophages are the predominant infiltrating immune cells in TME (75). Intriguingly, in early TNBC, F4/80^+^ Mϕs infiltrate the tumor, with half of them being FOLR2^+^ and CADM^-^ (69). Recent studies in the 4T1 orthotopic mouse model of TNBC showed the reprogramming of steady-state resident macrophages (referred as MGM). It led to altered cytokine signaling (TGFß, CSF-1, and IFN-gamma) mediated by specific transcription factors such as STAT1, RUNX3, and FOSL2 associated with poor outcome (43).

## 7 Breast adipose tissue and macrophage crosstalk in breast cancer

Adipose tissue stroma (ATS) is part of the breast anatomy and pathology. Large breast volume corresponds to high visceral fat (76) and is associated with worse outcomes in neoadjuvant chemotherapy compared to lean breast, especially in postmenopausal patients (77). In breast cancer, a lipid-associated macrophage (LAM) bearing a TREM2 signature is described, which is usually associated with monocytic origin (78). However, Dawson and colleagues described stromal Mϕs close to ATS (24). Moreover, another study in humans and mice showed two LAM populations by trajectory inference analysis: LAM-STAB1 and LAM-APOC1. Resident LAM-APOC1 was expressed both in the tumor and juxta-tumor area, strongly associated with CD8^+^ T cells and T-regs, while LAM-STAB1 was mostly expressed in the tumor and had a high level of TREM2 and IL-1B, suggesting their monocytic origin associated with poor prognosis (79). ATS and macrophages respectively release FFA and TNF-α in a paracrine manner to establish a vicious cycle to regulate each other (80). TRMs present antigens from dead adipocytes to attract recruited macrophages to form crown-like structures (CLS) in ATS, which increases aromatase activity, local invasion, and the possibility of metastasis (81).

## 8 Targeting resident macrophages for managing breast cancer

**Figure 2:**
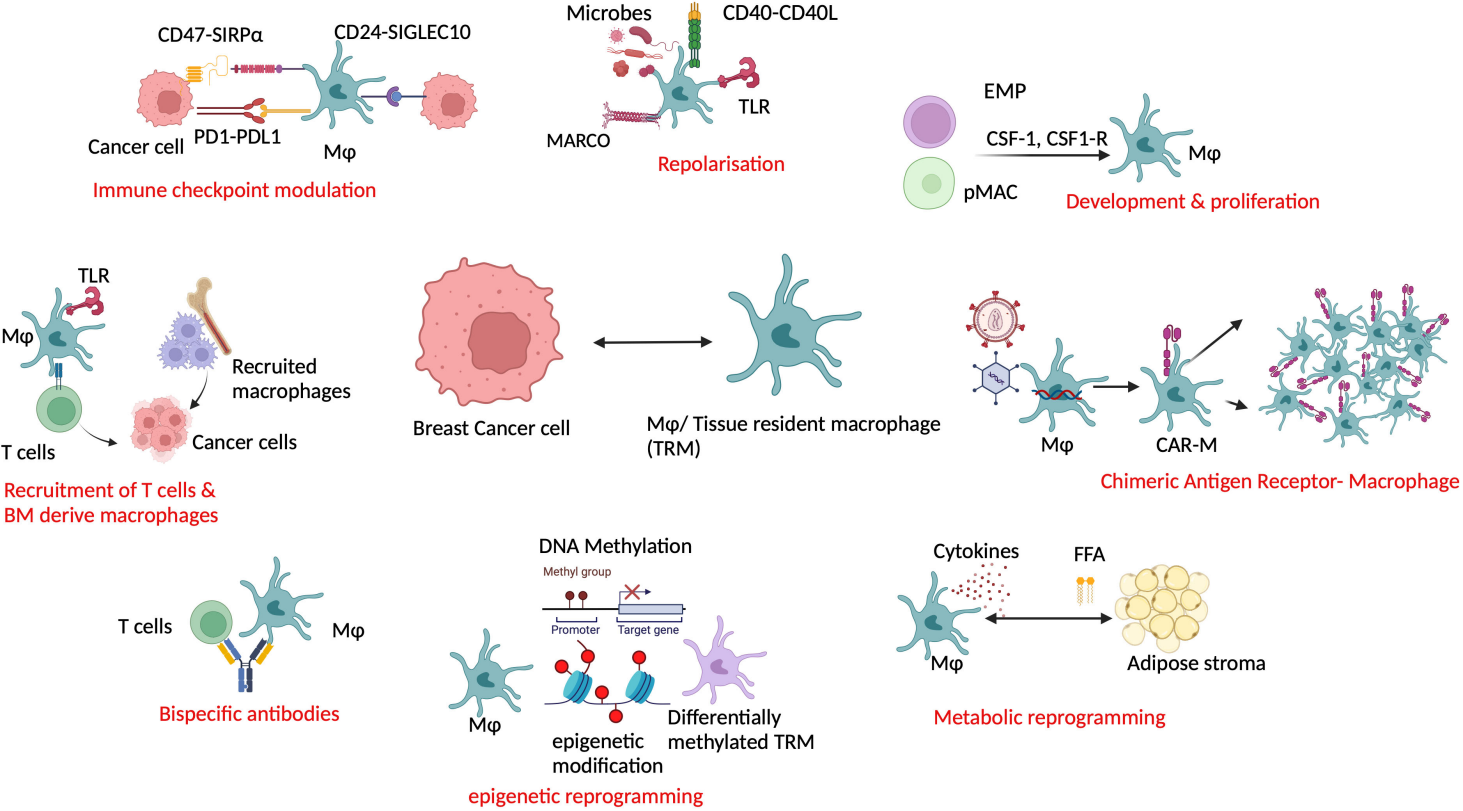
Co-targeting resident macrophage in breast cancer along with the current standard of care can be useful in advanced and refractory cases. Resident macrophages can be exploited in multiple ways such as by immune modulation, repolarization, reprogramming, recruitment, and as a drug delivery system in combination with other modalities. Mφ/TRM, tissue-resident macrophage; TLR, Toll-like receptor; MARCO, macrophage receptor with collagenous structure; BM, bone marrow; EMP, erythroid myeloid progenitor; pMac, pre-macrophage; CSF1, colony-stimulating factor 1; CSF1R, colony stimulating factor 1 receptor; CAR-M, chimeric antigen receptor macrophage; FFA, free fatty acid.

Targeting macrophages as a potential combination agent in breast cancer treatment is an area of active research. TRMs can be co-targeted based on their ability to interact with other cells, predominantly T cells and fibroblasts. As many available therapies are TAM centric, TRMs being the predominant part of TAMs (28, 69) are also targeted. TRMs inhibit cytotoxic T-cell activity in multiple ways like modulating checkpoints, cytokine release, impaired antigen presentation, and induction of collagen remodeling enzymes (82–84). In human BRCA-1-associated TNBC, PARP inhibitors (PARPi) increase infiltrating CD206^+^PDL-1^+^CSF1R^+^ macrophages, which are immunosuppressive to cytotoxic T cells. Combining CSF-1R inhibitors with PARPi for this group showed improved CD8^+^ T-cell-mediated survival in mice (75). Another possible combination with PARPi in breast to bone metastasis is zoledronate because it blocks osteoclasts, the local Mϕ in the bone. Osteoclasts reduce the sensitivity of Cisplatin and PARPi by increasing glutamine production (74). Also as half of the HER2^+^ tumors are immunogenic, targeting macrophage and T cells combined with bispecific antibodies or checkpoint inhibitors can be a therapeutic possibility in those patients (85, 86). Immune checkpoint inhibitors can be useful in targeting TRMs for two different reasons: first, their abundance in breast TME and, second, their ability to modulate CD8^+^ cytotoxic T cells and immune check points (55–57). Blocking CD47-SIRP-α axis to improve immunosuppression (48) and CD24-Siglec10 in TRMs to evade immune escape of tumor cells are evolving options (87). Blocking immunosuppressive macrophages may also improve the efficacy of immune checkpoint inhibitors. As discussed earlier, TAMs can confer chemoresistance through fibroblasts via CCL18-PITPNM3 signaling. Blocking CCL18–PITPNM3 signaling by inhibiting TAMs can prevent tumor progression, delay metastasis, and prevent immunosuppression (62). TRMs can be targeted by several other mechanisms supplementing SOC (**Figure 2**). Repolarization of the TRMs using small molecule inhibitors or microbes from pro-tumor TRMs to antitumor ones is one of the proposed models (88). Using macrophage as a drug delivery system such as chimeric antigen receptor macrophage therapy (CAR-M) (89) is gaining more attention recently. As of February 2024, two clinical trials testing CAR-M-based strategy for breast cancer are registered: NCT04660929 on HER2^+^ patients with refractory or relapsed disease (recruiting) and NCT05007379 (CARMA) on patient derived organoids (90, 91). However, whether resident macrophage as CAR-M has any additional pros is yet to be studied. Two specific mechanistic scenarios can be considered for targeting TRMs. TRMs can present antigen to the monocyte-derived macrophages and T cells, recruiting them to the breast TME. This can be further strategized for drug discovery. Furthermore, metabolic reprogramming of resident macrophages by adipose tissue stroma can be explored, as the crosstalk between the two is the guiding mechanism of metabolic diseases predisposing cancer. Finally, epigenetic reprogramming of resident macrophages such as TMP195 can be an attractive treatment option in the future (92). Current strategies targeting macrophages as single or combination agents are illustrated in **Figure 2**.

## 9 Discussion

Macrophages are the most abundant and transcriptionally diverse innate immune cells in the breast cancer microenvironment. Their cancer-specific reprogramming facilitates cancer progression and metastasis. However, a major challenge is to separate TRMs from recruited ones within the TAM pool. Such separation is required because they have different and often contradictory functions. For example, TAMs release CSF-1 and CXCL1 helping tumor migration and EMT (93, 94). Furthermore, the hypoxic environment created by TAMs and cancer cell crosstalk triggers NFACTc1-mediated osteoclastogenesis to make a circulating metastatic niche. In addition, TAM causes induction of HIF1a by NF-kB activation, which is implicated in the pathogenesis of breast-to-bone metastasis but by which TAM subpopulation, which is not entirely clear (95–97). One major challenge is the availability of very little imaging information regarding TRMs due to strong overlap of commonly used reporters between myeloid cell subsets. Developing different reporter mice can improve our knowledge of the resident TAMs and further target them (40). Recently, combining scRNA-seq data with spatial mapping using multiple transgenic fluorescent reporter mice revealed a massive increase in intraepithelial CD11b^−^ macrophages in breast TME, interacting with tumor cells at all stages of disease progression (30). These macrophages are ductal and found inside the tumor epithelium passing through the breached basement membrane (30). This is interesting, as these ductal TAMs correspond to the Mϕ3 discussed earlier and their fetal origin was confirmed by fate mapping studies using Ms4a3–Cre/Rosa–tdTomato mice (24). The second CD11b^+^ TAM population is monocyte derived and expressed in TREM2 and SPP1 (30, 98–100). Another stromal FOLR2^+^ and LYVE1^+^ TAM subset found in perivascular tissue niche are part of Mϕ TAMs (30, 101). Intriguingly, in human primary luminal breast cancer, two subsets of APOE^+^ TAMs are described by scRNA-seq, expressing either TREM2 or FOLR2 (60, 101). TREM2 and FOLR2 expressions determine the functional status and spatial distribution of TAMs. As TREM2^+^ macrophages are infiltrating in nature during cancer development and transcriptionally proximal to CD14^+^ CCR2^+^ monocytes in breast cancer, they are concluded as HSC-derived recruited macrophages (60, 101, 102). LYVE1^+^ FOLR2^+^ macrophages found in breast TME are perivascular (101). Studies in mice and the human brain showed that perivascular macrophages, although having a postnatal developmental switch, reside in the CNS without any contribution from HSC-derived precursors (103–105) suggesting their residential nature. Indeed, scRNA-seq analysis of mice macrophages compared with a publicly available database of human macrophages and genetic fate mapping confirms that the FOLR2^+^ TIMD4^+^ LYVE1^+^ macrophages are self-maintaining Mϕs (106). Finally, a SIGLEC-1^+^TAM is described in human breast cancer associated with aggressive subtypes and shorter survival; however, their developmental origin remains transcriptionally unique (46). Therefore, a clear developmental diversity exists in the breast TAMs. Initial immunotherapy trials for breast cancer were directed toward T cells, but the response was limited, e.g., in JAVELIN (NCT01772004) and KEYNOTE-028 (NCT02054806) (107). However, a bispecific approach with PD1-IL2v to expand stem cells like CD8^+^ T cells and anti-PD-L1 to reprogram macrophages and vasculature in immunotherapy-resistant pancreatic neuroendocrine tumors in RIP1-Tag5 mouse model showed complete tumor regression (6). Clinical.trial.gov database search using keywords “macrophage” and “breast cancer” showed 81 trials having macrophages in combination with hormonal therapy, chemotherapy, or immunotherapy until January 24. Toward this goal, a recent study isolated five TRM clusters from breast cancer patients by analyzing scRNA-seq data (108). This signature database can help make informed combinatorial treatment decisions by cotargeting TRMs alongside SOC. Moreover, the FOLR2^+^ TRMs are shown to promote T-cell infiltration in the tumor, thereby increasing immunogenicity and antitumor activity (108). Therefore, combination with immune checkpoint inhibitors and FOLR2^+^ TRM promoters can help in resistant cases.

## 10 Conclusion

In a straightforward scenario, resident macrophages maintain homeostasis, and recruited macrophages would promote inflammation in breast TME. However, realistically, the reprogramming of resident macrophages confers additional layers of heterogeneity challenging the therapeutic development. Better spatiotemporal delineation of macrophage niche in TME and identifying reprogramming mechanisms may identify dynamic cellular states rather than rigid phenotypes. Understanding the TRM heterogeneity will pave the way for novel targets and potential combinations.

## Author contributions

MB: Conceptualization, Data curation, Investigation, Methodology, Project administration, Resources, Software, Visualization, Writing – original draft, Writing – review & editing.
